# ROHHAD Syndrome, a Rare Cause of Hypothalamic Obesity: Report of Two Cases

**DOI:** 10.4274/jcrpe.0027

**Published:** 2018-11-29

**Authors:** Ülkü Gül Şiraz, Deniz Ökdemir, Gül Direk, Leyla Akın, Nihal Hatipoğlu, Mustafa Kendirci, Selim Kurtoğlu

**Affiliations:** 1 Erciyes University Faculty of Medicine, Department of Pediatric Endocrinology, Kayseri, Turkey

**Keywords:** ROHHAD syndrome, hypothalamic dysfunction, endocrinological disorders

## Abstract

Rapid-onset obesity with hypoventilation, hypothalamic dysfunction and autonomic dysregulation (ROHHAD) syndrome is a rare disease that is difficult to diagnosis and distinguish from genetic obesity syndromes. The underlying causes of the disease have not been fully explained. Hypothalamic dysfunction causes endocrine problems, respiratory dysfunction and autonomic alterations. Currently there are around 80 reported patients although this is likely due to underdiagnosis due to lack of recognition. We present two female patients suspected of ROHHAD due to weight gain starting in early childhood. Clinical and biochemical findings such as respiratory and circulatory dysfunction, hypothalamic hypernatremia, central hypothyrodism, hyperprolactinemia and central early puberty in these patients matched the criteria for ROHHAD syndrome. ROHHAD syndrome should be considered in the differential diagnosis of monogenic obesity.

**What is already known on this topic?**

Rapid-onset obesity with hypoventilation, hypothalamic dysfunction and autonomic dysregulation syndrome is a rare disease. There are around 80 reported patients.

**What this study adds?**

Rapid-onset obesity with hypoventilation, hypothalamic dysfunction and autonomic dysregulation (ROHHAD) syndrome should be considered in the differential diagnosis of obese patients with hypothalamic dysfunction and autonomic alterations. We aimed to increase awareness about ROHHAD syndrome which does not have dysmorphological features with obesity.

## Introduction

Rapid-onset obesity with hypoventilation, hypothalamic dysfunction and autonomic dysregulation (ROHHAD) syndrome is a rare cause of obesity, characterized by early and rapid onset of obesity, hypoventilation, hypothalamic dysfunction and autonomic dysfunction. To date, 80 patients with ROHHAD have been reported (1,2,3). Obesity and endocrine abnormalities comprise major components of the syndrome. It should therefore be kept in mind in the differential diagnosis, particularly of pediatric endocrinology patients.

The syndrome begins with an uncontrollable eating impulse and rapid weight gain starting in early childhood, usually around 2-4 years of age. In this period, episodes of apnea and cyanotic attacks, due to respiratory distress caused by weight gain and hypothalamic dysfunction, are clinically apparent. Hyponatremia and/or adipsic hypernatremia may also be seen due to involvement of the thirst center. Abnormalities of hypothalamo-hypophyseal hormones may lead to presentation with diabetes insipidus, hyperprolactinemia, growth hormone (GH) deficiency, central hypothyroidism, secondary adrenal insufficiency and/or early or delayed puberty (2,4,5).

There may be findings of autonomic dysfunction including thermal dysregulation (hypothermia/hyperthermia), cold, pale hands and feet (Raynaud’s phenomenon), excessive sweating, decreased pain sensitivity, impaired pupillary response to light, bradycardia, hypotension and gastrointestinal dysmotility (2,6,7). Moreover, patients may present with psychiatric problems such as anxiety, aggressive behavior and personality disorder (8). The majority of patients were reported to have an underlying neural crest tumor (5). Thus, this rare syndrome can manifest with various clinical and endocrine findings.

In this paper, we discuss two cases with distinct clinical symptoms who presented for evaluation of obesity.

## Case Reports

### Case 1

A 7 years 4 months old girl presented with excessive weight gain. She was born at term with a birth weight of 2900 g [-0.96 standard deviation (SD)]. The parents reported that she began to gain weight rapidly at 1.5 years of age. There was no consanguinity between the parents. The patient had two healthy siblings (of ages 11 years and 9 months). Body weight was 61 kg (±3.9 SD) and height was 130 cm (±1.8 SD) with a body mass index (BMI) of 36 kg/m2 (±3.3 SD). She had a plethoric facial appearance, axillary acanthosis nigricans, pale/blue fingers and toes and stage 2 thelarche, bilaterally. The patient was admitted to hospital for further evaluation. During follow-up, it was observed that she had episodes of excessive sweating and a body temperature as low as 35.4 °C. Blood pressure was 95/60 mmHg (95 percentile: 120/80 mm/Hg). Laboratory evaluation showed the following results: sodium (Na), 156 mmol/L [normal range (NR): 135-145 mmol/L]; aspartate transaminase, 87 U/L (NR: 8-45 U/L); alanine aminotransferase, 57 U/L (NR: 7-55 U/L); urine density, 1024. The remaining liver function tests, serum electrolytes, lipids kidney function tests and complete blood count were normal. The patient had no polydipsia; thus, hypernatremia was considered to be due to insufficient intake. Oral fluid replacement was given and the hypernatremia was corrected (Na: 141 mmol/L). Impaired glucose tolerance (141 mg/dL at two hours) was detected in the oral glucose tolerance test performed due to morbid obesity and acanthosis nigricans; and the patient was started on metformin. An abdominal ultrasound was performed, due to the elevated transaminase levels, which revealed grade 3 hepatic steatosis.

In terms of hormonal problems that may present in the patient’s follow-up hormonal evaluation revealed the following results: free T4, 0.7 ng/mL (NR: 0.98-1.6 ng/mL; thyroid stimulating hormone (TSH), 4.8 µIU/mL (NR: 0.5-4.3 µIU/mL); adrenocorticotropic hormone (ACTH), 24 pg/mL (NR: 10-60 pg/mL); cortisol, 1.5 µg/mL (NR: 3-21 µg/mL); LH, 1.3 mIU/mL (prepubertal NR: <0.3 mIU/mL); estradiol, 12.9 pg/mL (prepubertal NR: <12 pg/mL); prolactin (PRL), 33 ng/mL (NR: 4.7-23.3 ng/mL). Insulin like growth factor-1 (IGF-1) was <25 ng/mL and IGF binding protein 3 (IGFBP3) 1870 ng/mL. Bone age was advanced at 10 years. Peak cortisol response to low dose ACTH stimulation test was low (9.9 µg/mL).

**Figure 1:**
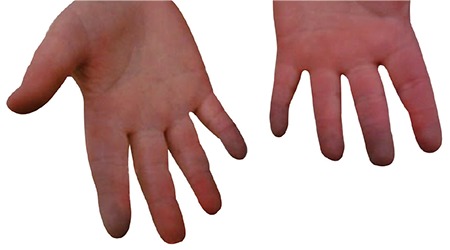
Raynaud’s phenomenon in case 1

A diagnosis of secondary adrenal insufficiency, central hypothyroidism and central precocious puberty was made and treatment was initiated with hydrocortisone, thyroxine and leuprolide acetate. No further evaluation or treatment was considered for GH deficiency, due to the patient’s normal height. However, a predisposition to neural crest tumor was considered despite the low IGF-1 and IGFBP3 levels of the patient. Brain and pituitary magnetic resonance (MR) imaging studies were found to be normal. The IQ score was 65. The pale appearance of the fingers was considered to be due to Raynaud’s phenomenon (Figure 1). Alterations in body temperature and Raynaud’s phenomenon were attributed to autonomic dysfunction. Pulmonary hypertension was detected on echocardiography and nifedipine was prescribed.

### Case 2

**Figure 2:**
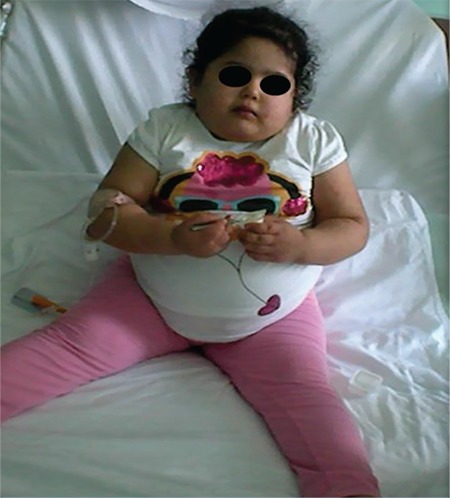
Appearance of case 2

A five year old girl with suspected epileptic seizures was referred for evaluation of obesity. The patient was reported to have sleep apnea and aggressive behavior. She was born at term, with a birth weight of 2800 g (-1.2 SD) and she had uncontrollable eating, starting at two years of age, with rapid weight gain. There was no consanguinity between parents and she had two healthy siblings. Body weight was 11 kg (±3.7 SD) and height was 101 cm (±1.7 SD) with a BMI of 30.4 kg/m^2^ (±5.7 SD) (Figure 2). The patient had central cyanosis. Blood pressure was 90/60 mm/Hg (95 percentile: 115/75 mm/Hg). Axillary body temperature measurements varied from 35.6 to 39.5 °C.

Laboratory evaluation revealed the following results: Na, 164 mmol/L (NR: 135-145 mmol/L); urine density, 1018. The remaining biochemical parameters including liver enzyme levels and lipid profile were normal. The patient was considered as a case of adipsic hypernatremia. Oral fluid replacement was given, which normalized the Na value (140 mmol/L). Pituitary evaluation revealed the following results: free T4: 0.8 ng/mL (NR: 0.98-1.6 ng/mL); TSH: 1.8 µIU/mL (NR: 0.5-4.3 µIU/mL); PRL: 56 ng/mL (NR: 4.7-23.3 ng/mL). Remaining pituitary hormone levels were within normal limits.

Treatment was started for central hypothyroidism. The mild hyperprolactinemia persisted but no treatment was needed. Brain and pituitary MR imaging studies revealed normal results. Genetic tests for the Prader-Willi syndrome revealed no abnormality in the 15q11-q13 (*SNRPN* gene). In addition, the genetic analysis did not identify any abnormality in the gene associated with congenital hypoventilation syndrome, *PHOX2B*. IQ was compatible with a chronological age of 3 years. Imaging studies for neural crest tumor gave normal results. However, sleep apnea persisted and the central cyanosis progressed. The patient was transferred to the intensive care unit due to development of carbon dioxide retention (pH: 7.27, pCO_2_: 55 mm/Hg) and mechanical ventilation was initiated. Since the patient needed continuous ventilator support, she was discharged with a home ventilator after tracheostomy.

The clinical and laboratory characteristics of the two patients are summarized in Table 1.

## Discussion

ROHHAD syndrome, characterized by high morbidity and hypothalamic dysfunction is a rare cause of obesity with unknown etiology (7). It has been suggested that the syndrome is associated with underlying genetic, autoimmune and paraneoplastic factors and studies aiming to detect such factors are ongoing (4). However, in one such study investigating candidate autonomic system genes no genetic defect was found (9).

In the Smiths-Magenis syndrome (SMS), presenting in late childhood/adolescence with hyperphagia and dysmorphism, a point mutation in *RAI1*, which is a transcription factor involved in craniofacial and neural development, is implicated (10). In 2015, a novel mutation was detected in the *RAI1* gene in a boy aged 11 years who presented with the clinical findings of ROHHAD syndrome and the authors suggested that the patient had an overlap syndrome with SMS (11).

Since patients with ROHHAD syndrome resemble congenital hypoventilation syndrome patients, *PHOX2B*, a gene encoding a transcription factor critical for hypothalamic embryogenesis, was investigated but no abnormalities were found (9). Congenital hypoventilation syndrome manifests with autonomic dysfunction, respiratory problems and gastrointestinal motility disorders in the neonatal period in most instances. In both our patients, onset of obesity was after 18 months of age and autonomic dysfunction developed later in the course of the disease.

Prader-Willi syndrome is also included in the differential diagnosis of these cases, due to early onset of obesity. Mental retardation, hypotonia, small hands and feet, ocular findings and hypogonadism may also be present (2,10,12). Prader-Willi syndrome was not considered in the differential diagnosis of the first case due to the presence of precocious puberty. A genetic investigation was performed in the second case, which proved to be negative.

Leptin deficiency or resistance can be distinguished from ROHHAD syndrome by earlier onset of weight gain. This condition is associated with immune deficiency, but also causes hyperphagia, obesity and deficiency in pituitary hormones. MRC4 receptor resistance - proopiomelanocortin (POMC) deficiency is also a rare cause of monogenic obesity but in these patients obesity begins in the first year of life and no autonomic dysfunction is described. In addition, there is usually reddish hair and extremely light skin color in POMC deficiency (13,14).

Cushing syndrome should be kept in mind in the differential diagnosis of patients with ROHHAD syndrome. No hypercortisolism was detected in either of our patients; indeed secondary adrenal insufficiency was present in the first case.

Autonomic dysfunction is one of the common findings in patients with ROHHAD syndrome. Tachycardia/bradycardia, cardiac arrest, constipation, hypothermia/hyperthermia, sleep apnea and narcolepsy may be present. Dysfunction of hypocretin-1, involved in acetylcholine release in the autonomic nervous system, has been implicated in autonomic dysfunction (7). Hypothermia, excessive sweating and Raynaud’s phenomenon were present in the first case, while there were hypothermia-hyperthermia episodes and sleep apnea in the second case. Respiratory problems and obstructive sleep apnea can be observed in other monogenic obesity cases but there are no additional clinical findings such as thermal dysfunction, excessive sweating and circulatory problems such as Raynaud’s phenomenon. In addition adipsic hypernatremia has not been reported in other monogenic obesity cases.

The presence of lymphocytic infiltration on postmortem hypothalamic examination is suggestive of autoimmunity in ROHHAD syndrome and a partial response has been reported to intravenous immunoglobulin and immunosuppressive therapies (5,12). In addition, detection of an oligoclonal band in the cerebrospinal fluid analysis supports intrathecal immunoglobulin synthesis (12).

Ganglioneuroma and neuroblastoma have been reported in some ROHHAD cases, suggesting paraneoplastic involvement of the hypothalamus (2). Thus, there is a group of patients comprising 40% of all patients, the term “ROHHAD-neuroendocrine tumors” has been coined in recent years (15). We screened both cases for the presence of such tumors but no mass lesion was detected.

ROHHAD syndrome is a rare cause of hypothalamic obesity and is accompanied by autonomic dysfunction and pituitary hormone abnormalities. It is a multi-systemic disease with unclear etiology, requiring a multidisciplinary palliative approach. It is thought that the diagnosis is missed in many of these cases, most of whom die due to respiratory or cardiac problems or to an underlying neoplasm. ROHHAD syndrome should be kept in mind in the differential diagnosis of monogenic obesity.

## Figures and Tables

**Table 1:**
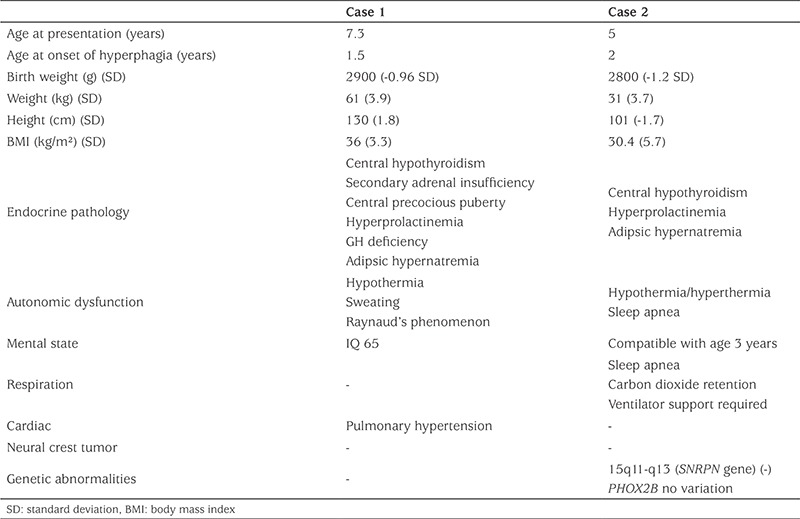
Clinical and laboratory features in the two patients
